# Venous Malformations as a Rare Cause of Knee Joint Pain in Children and Young Adults: Clinical and Radiological Manifestations

**DOI:** 10.3390/children12040514

**Published:** 2025-04-16

**Authors:** Adam Dobek, Marcin Strzelczyk, Ludomir Stefańczyk, Dobromiła Barańska, Jan Sokal, Przemysław Przewratil

**Affiliations:** 1 I Department of Radiology and Diagnostic Imaging, Norbert Barlicki Memorial Teaching Hospital No. 1, Medical University of Łódź, 90–419 Łódź, Poland; 2 Department of Radiology, Maria Konopnicka Memorial Teaching Hospital, 91-738 Łódź, Poland; 3 Department of Diagnostic Imaging, Polish Mother’s Memorial Hospital Research Institite, 93-338 Łódź, Poland; 4 Department of Pediatric Surgery and Oncology, Maria Konopnicka Paediatric Centre Medical University of Łódź, 91-738 Łódź, Poland

**Keywords:** intra-articular, joint, knee, magnetic resonance imaging, venous malformations

## Abstract

**Background**: Venous malformations (VMs) are congenital vascular abnormalities characterized by tortuosity, slow blood flow, and gradual growth. Intra-articular venous malformations (IAVMs) of the knee are rare and often present with symptoms similar to juvenile idiopathic arthritis (JIA) or late sequelae of trauma. VM in children is commonly misdiagnosed as hemangioma. This study aims to analyze the clinical and MRI features of IAVM in the knee joint. **Methods**: This retrospective study analyzed patients from a reference unit for the treatment of vascular malformations in the Pediatric Surgery Department. The group was collected starting from the year 2014 until the 100th patient was identified in the year 2018, all with MRI-confirmed VM based on a predefined protocol. From this group, 19 patients with lower limb symptoms were identified, and 9 patients with VM involving the knee joint were selected for further analysis. **Results**: The most common symptoms in IAVM patients were pain and swelling, chronic in five (55%) and intermittent in four (45%). Four (45%) reported worsening pain during or after physical activity. A history of intra-articular bleeding was noted in five (55%), leading to mild knee contracture (10° reduction in extension) and decreased mobility. Limb deformities were observed in eight (89%). Diffuse VMs, affecting both intra- and extra-articular tissues, were present in eight (89%), involving the thigh in seven (78%), crus in five (56%), gluteal muscles in three (33%), and foot tissues in one (11%). The suprapatellar recess and Hoffa’s fat pad were involved in all patients (100%). **Conclusions**: IAVMs are rare causes of knee dysfunction in children and young adults, particularly in cases of unexplained pain, swelling, or instability. They should be considered in the differential diagnosis of hemophilic arthropathy, JIA, or late post-traumatic sequelae. Untreated IAVMs can lead to intra-articular bleeding, cartilage degeneration, and disability. Early diagnosis via MRI and ultrasound is crucial to identifying IAVMs and preventing joint degeneration. Timely treatment helps avoid further damage and long-term disability.

## 1. Introduction

Venous malformations (VMs) are congenital vascular anomalies characterized by tortuosity, slow blood flow, and reduced venous wall thickness. These lesions grow in tandem with the patient and do not regress spontaneously. Clinical symptoms typically manifest in early childhood, although they may remain asymptomatic until adulthood. The clinical presentation varies considerably depending on the malformation’s location and size. Histologically, VMs exhibit an absence or marked reduction in the muscle layer of the venous walls. Although these malformations may be located superficially—appearing as varicosities or capillary malformations on the skin surface—they frequently extend into deeper tissues and organs. In some patients, local intravascular coagulopathy (LIC) occurs, resulting in increased D-dimer levels and hypofibrinogenemia. In rare cases, VM can occur intra-articularly (IAVM), leading to joint pain, swelling, and hemarthrosis, which may eventually result in joint damage. These symptoms are often misdiagnosed as other conditions, such as juvenile idiopathic arthritis (JIA) or the late sequelae of trauma [1,2].

Furthermore, due to the inaccurate use of terminology, vascular malformations are frequently not differentiated from vascular tumors, such as hemangiomas, which in pediatric patients are most commonly of the infantile type. In contrast, vascular tumors are characterized by rapid growth, a subsequent developmental plateau, and gradual regression [3]. Accurate classification of vascular anomalies and tumors is essential, as misclassification can delay appropriate treatment. The initial classification of vascular anomalies was published by Mulliken and Glowacki in 1982 [4] and has since been refined with the identification of causal genetic mutations. The most recent revision was made by the International Society for the Study of Vascular Anomalies (ISSVA) in May 2018 [5]. This classification divides vascular anomalies into vascular tumors (benign, locally aggressive or borderline, and malignant) and vascular malformations, which are further categorized as either simple or combined malformations (the latter being defined as lesions containing two or more types of vascular malformations).

Simple malformations include capillary malformations, lymphatic malformations, arteriovenous malformations (AVMs), arteriovenous fistulas (AVFs), and VMs, which are the focus of this study. The most common vascular malformations are VMs, which are further subtyped into common VM, familial cutaneo-mucosal VM, blue rubber bleb nevus (Bean) syndrome VM, glomuvenous malformation, cerebral cavernous malformation, familial intraosseous vascular malformation, and verrucous venous malformation. The diagnostic process for VM begins with a comprehensive clinical evaluation, followed by radiological imaging. Patients suspected of having a VM should undergo routine imaging, including color Doppler ultrasonography (US) and MRI, which are the preferred methods for assessing lesion extent. These imaging techniques are crucial for differentiating vascular malformations from vascular tumors and for distinguishing between fast-flow and slow-flow malformations.

Once a diagnosis is confirmed and the characteristics of the lesion are determined, treatment options can be considered. Symptom management typically involves analgesics, compression therapy with elastic stockings, and low-molecular-weight heparin, especially in the presence of localized intravascular coagulopathy (LIC). For more extensive or symptomatic malformations, invasive treatments such as percutaneous sclerotherapy, laser therapy, or surgical intervention may be necessary.

Management of IAVM often requires a multidisciplinary approach, including radiologists, pediatric surgeons, orthopedic specialists, and vascular surgeons [6,7,8]. VMs of the knee joint are rare, but it is crucial to consider them in the differential diagnosis of knee disorders in children and young adults. If left untreated, VM can result in significant pathological changes in the knee. This manuscript aims to raise awareness of this pathology and highlight its clinical and radiological manifestations, helping clinicians more effectively recognize and manage these conditions.

## 2. Materials and Methods

### 2.1. Patient Identification and Inclusion Criteria

This retrospective study analyzed patients from a reference unit for the treatment of vascular malformations in the Pediatric Surgery Department. The cohort was assembled from December 2014 until the 100th patient was identified in October 2018. The inclusion criteria were contrast-enhanced MRI-confirmed VM, based on a predefined protocol and in accordance with ISSVA guidelines. From this group, 19 patients with lower limb VMs and documented symptoms in their medical histories were identified. These symptoms included a history of hemarthrosis, chronic pain, limb deformity, and joint contracture in the affected limb. The final study group was selected based on the location of the malformations in the knee joint area.

### 2.2. Magnetic Resonance Imaging Protocol

The MRI protocol for imaging vascular malformations may vary among patients; however, it should include multiple sequences with T1-, T2-, and STIR-weighted imaging. The use of an intravenous contrast agent (gadolinium) is essential for differentiating VMs from other vascular anomalies, such as lymphatic malformations. An STIR- or T2-weighted sequence with fat saturation in the coronal or sagittal plane is performed to cover a large area of interest and delineate the extent of the lesion. A T1-weighted sequence in the axial plane is used to assess muscle involvement and detect high T1 signal contents within the VM, such as blood, proteinaceous material, or fat. Additionally, an STIR- or T2-weighted sequence with fat saturation in the axial plane aids in evaluating lesion morphology and its relationship with adjacent structures, including the skin, neurovascular bundles, and bone. Gradient-recalled echo (GRE) sequences may reveal blooming artifacts, which are suggestive of internal calcifications or phleboliths. Pre- and post-contrast T1-weighted sequences with fat saturation in at least two perpendicular planes enable the assessment of enhancement patterns in dynamic imaging. Contrast-enhanced MR angiography is valuable for evaluating flow dynamics, diagnosing high-flow malformations, detecting AVF, and assessing residual flow in lesions following treatment [9]. Optionally, a T1-weighted sequence with fat saturation after gadolinium administration in the late venous phase, acquired in at least two perpendicular planes, may demonstrate delayed enhancement in low-flow venous malformations. This allows for the differentiation between venous and lymphatic components in mixed malformations. All MRI studies were conducted using a Philips Ingenia 1.5T MRI scanner (Philips Healthcare, Best, The Netherlands).

### 2.3. Final Patient Selection for Data Analysis

The analysis encompassed both clinical features and radiological findings. Clinical features included a history of hemarthrosis, chronic pain, limb deformity, joint contracture in the affected limb and the severity of these symptoms. Radiological features were also thoroughly assessed, focusing on the localization of lesions (intra-articular or both intra- and extra-articular), involvement of extracapsular structures (subcutaneous tissue, muscles, and ligaments), and the presence of VM in Hoffa’s fat pad and joint recesses.

## 3. Results

### 3.1. Patients

Contrast-enhanced MRI revealed intra-articular involvement of VM in nine patients. The study group consisted of five males aged 10 to 22 years (mean age: 16) and four females aged 3 to 21 years (mean age: 12).

### 3.2. Clinical Symptoms

The most common symptoms reported by all patients diagnosed with IAVM were pain and swelling. These symptoms were chronic in five (55%) cases and intermittent in four (45%). Four patients (45%) experienced worsening pain during or after physical activity. A history of intra-articular bleeding was reported in five patients (55%), leading to joint contracture and reduced mobility of the affected limb. However, the degree of knee contracture was generally mild, with full extension reduced by approximately 10 degrees on average. Limb deformity was observed in eight patients (89%) (Table 1).

### 3.3. Radiological Features of VM of the Knee on Contrast-Enhanced MRI

**Figure 1:**
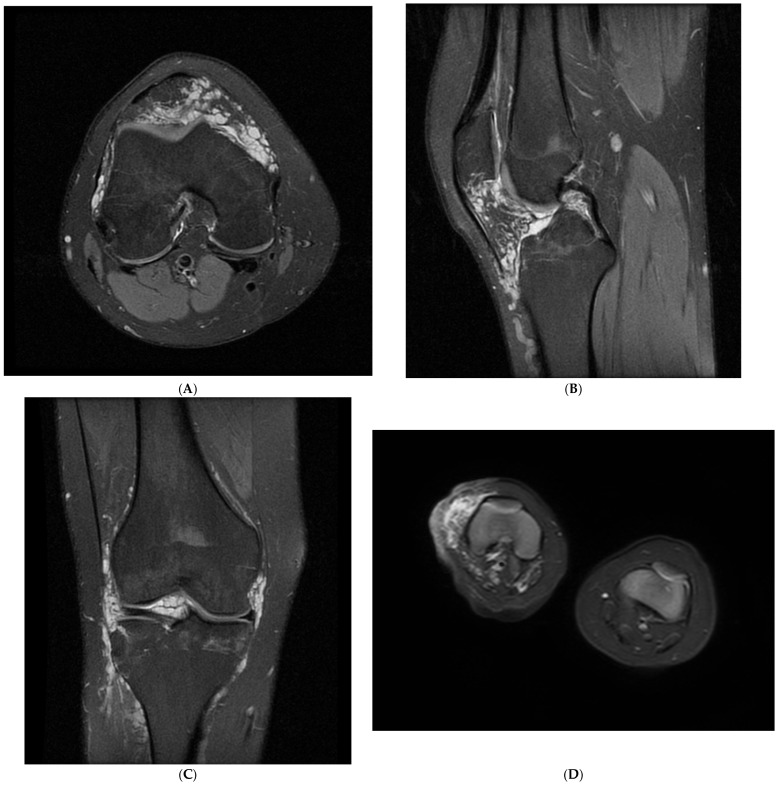

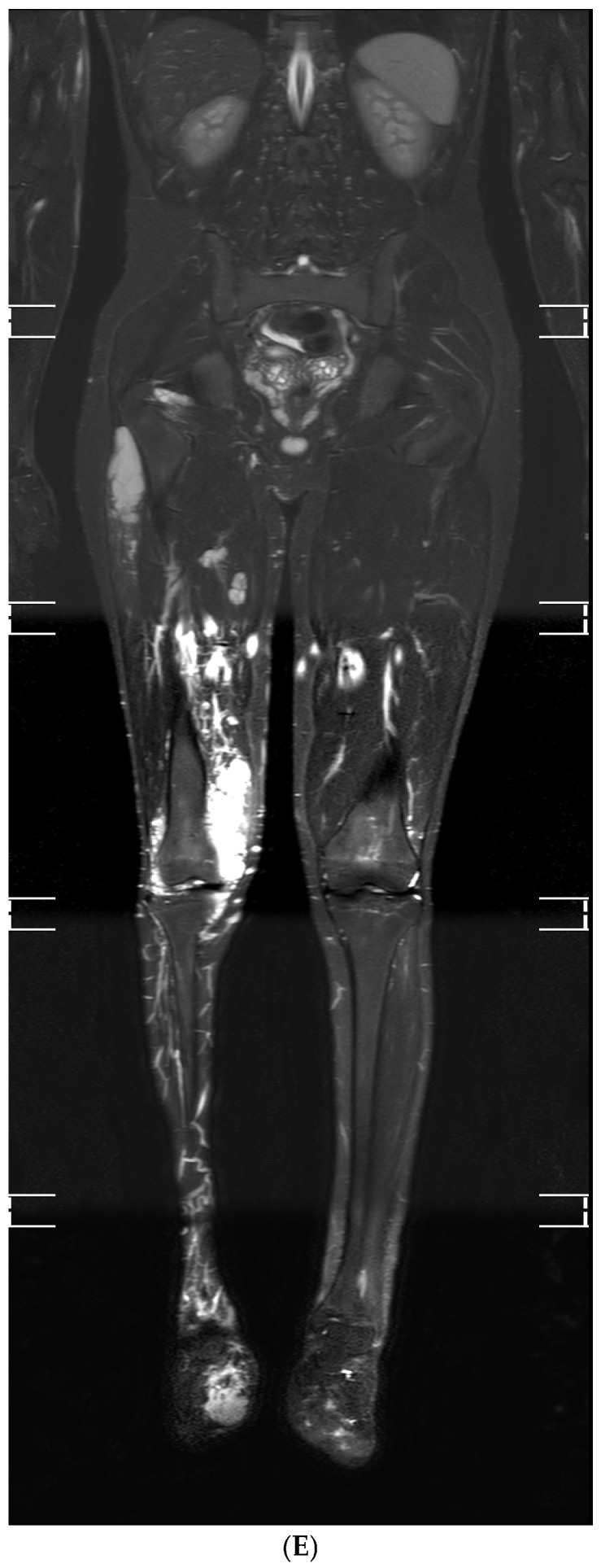
(**A**) PD-weighted image with fat saturation in axial plane demonstrating intra-articular venous malformation encompassing distal femoral epiphysis, (**B**) PD-weighted image with fat saturation in sagittal plane demonstrating intra-articular venous malformation involving infrapatellar (Hoffa’s) fat pad, (**C**) PD-weighted image with fat saturation in coronal plane demonstrating intra-articular venous malformation involving knee joint, (**D**) T2-weighted image with fat saturation comparing venous malformation involving right knee joint with normal tissues of left lower extremity, (**E**) T2-weighted STIR image showing venous malformation involving entire right lower extremity.

Only one patient in the study group was diagnosed with a VM confined solely to the knee joint. Eight patients (89%) had diffuse VMs of the knee, involving both intra- and extra-articular tissues. The affected extra-articular structures included the thigh muscles (seven patients (78%)), crus muscles (five patients (56%)), and gluteal muscles (three patients (33%)). In one case, the tissues of the foot were also involved (11%). The suprapatellar recess and Hoffa’s fat pad were affected in all patients (100%) (Table 2) (Figure 1A–E).

## 4. Discussion

From a selected group of 100 patients diagnosed with VM via contrast-enhanced MRI, 19 patients were identified in whom the malformations involved the lower extremities. Of these, nine had VM that involved the knee joint. The knee is the most commonly affected joint in VM, accounting for approximately 60% of all cases [10]. Symptoms such as unilateral knee pain and swelling typically appear in childhood and can mimic other conditions, including JIA, pigmented villonodular synovitis, or various tumors. All of our patients presented with pain and swelling in the affected limb; however, the nature of the pain, including its type, onset, and timing, varied among them. Additionally, all patients exhibited some form of limb deformity. Intra-articular bleeding, the most significant complication associated with the presence of IAVM, was observed in approximately half of the patients. This mirrors the situation in severe hemophilia, where recurrent hemarthrosis is the most common clinical manifestation. If not properly managed, even mild hemarthrosis can lead to hemophilic arthropathy—a debilitating condition characterized by joint deformation, chronic pain, and significant impairment in quality of life, which may ultimately require joint replacement [11]

Radiological evaluation in patients with suspected VM typically begins with a knee X-ray, which may reveal phleboliths in cases of VM. However, in most other instances, the findings are generally unremarkable [7]. Well-circumscribed, exclusively intra-articular VMs are rare, as most vascular anomalies present as extensive lesions affecting large portions of extra-articular tissues in the lower extremity [1,6,12]. US is widely used as an initial imaging modality due to its noninvasive nature, low cost, availability, and lack of ionizing radiation, the last of which is especially important in the pediatric population [8,13]. According to a review by Legiehn and Heran on grayscale imaging, VMs commonly appear heterogeneous in 98% of cases. The lesions are typically hypoechoic (82%) when compared to the surrounding tissue, though they may also be hyperechoic (10%) or isoechoic (8%). Vascular channels, represented by tubular anechoic structures, are seen in a minority of cases (4–50%). The phlebolith, a defining feature, is identified as a hyperechoic focus with acoustic shadowing, though it is present in only 16% of cases. When the lesion is close to the skin, it is often compressible. In some instances, the US finding may be limited to isoechoic skin thickening without a distinct mass or vascular channels. Doppler imaging, including color and pulsed Doppler, reveals vascular flow in 84% of lesions, with 78% exhibiting monophasic flow and 6% biphasic flow. No detectable flow is seen in 16% of cases, which may indicate thrombosis or flow below the detectable threshold. High-flow AVM can usually be differentiated from VM by the presence of enlarged vascular channels and the absence of a well-defined soft tissue mass. Doppler analysis of AVM shows high vessel density, increased systolic flow, and arteriovenous shunting [8]. In our view, the primary advantage of US lies in its capacity to evaluate both the velocity and characteristics of flow within the malformation, as well as to detect the presence of AVF, making it an indispensable tool in the diagnosis of VM.

However, in pediatric patients presenting with unexplained knee pain or suspected VM, MRI should be regarded as the preferred imaging modality for the diagnosis of vascular anomalies. This recommendation is based on the inherent limitations of US, including its relatively low spatial resolution, limited field of view—which may fail to encompass the entire lesion—and its inability to effectively assess deep, osseous, or periosteal structures [14]. Laboratory tests, including elevated D-dimer levels and hypofibrinogenemia, can assist in the diagnostic process. MRI enables assessment of lesion extent, preoperative planning, and postoperative monitoring, as well as visualization of residual VM and potential complications [1,7,8,9,15]. Typical MRI findings in VMs include tortuous, dilated tubular structures, often accompanied by a mass or soft tissue component. These lesions show high signal intensity on T2-weighted images and intermediate to low signal intensity on T1-weighted images. Occasionally, a high T1-weighted signal may be present due to fat or hemorrhage. Signal voids on T1- and T2-weighted sequences, as well as GRE imaging, correspond to calcifications and phleboliths, aiding in diagnosis. Differentiation between VMs and lymphatic malformations is possible based on contrast enhancement, as lymphatic malformations typically do not enhance. However, microcystic lymphatic malformations can be difficult to distinguish from VMs due to their homogeneous appearance and enhancement of cystic walls [8].

In contrast to vascular malformations, vascular tumors such as hemangiomas usually present as solid lesions with high T2-weighted signal intensity, along with prominent feeding arteries and draining veins [7]. A significant limitation of MRI, particularly in pediatric patients, is the requirement for complete immobility during the procedure. In young children, achieving this without general anesthesia is often problematic. Additionally, MRI is contraindicated in patients with claustrophobia or metal implants, further restricting its use. Although radiation-based imaging is generally avoided in pediatric populations, computed tomography (CT) may be considered when MRI is not feasible. A recent advancement in diagnostic imaging involves integrating conventional CT with electron density reconstructions, which can be particularly valuable for assessing complex malformations when MRI is contraindicated. This approach enhances tissue contrast and improves the visualization of subtle vascular structures, aiding in the differentiation of conditions such as hemophilic arthropathy, juvenile idiopathic arthritis, and post-traumatic sequelae [16].

The ISSVA classification should always be referenced when diagnosing vascular anomalies [17,18]. Misdiagnosis is common, as well-demarcated vascular malformations are often mistaken for vascular tumors, such as infantile hemangiomas, which are treated with propranolol instead of invasive therapy. Vascular malformations are congenital and generally grow proportionally with the patient, unlike vascular tumors, which exhibit rapid proliferation. While biopsy can aid in differentiation, it is not always necessary. Contrast-enhanced MRI and color Doppler ultrasound are often effective in identifying the lesion type, and if imaging suggests a characteristic VM, a biopsy may be avoided. High-flow lesions, such as AVM or AVF, can also be diagnosed through imaging, as biopsy may pose a bleeding risk [19]. In the authors’ opinion, performing a biopsy in cases of IAVM of the knee increases the risk of intra-articular bleeding. Since diagnosis can often be made through imaging alone, avoiding biopsy helps minimize unnecessary complications.

Our study indicates that the involvement of the suprapatellar recess and Hoffa’s fat pad, along with characteristic clinical symptoms, suggests intra-articular VM involvement. Similar findings have been reported by other authors, who note that MRI-based identification of synovial involvement may be limited by imaging resolution [1,2,7]. Diffuse vascular malformations affecting large areas of extra-articular tissue require extensive surgical intervention and additional minimally invasive treatments. Pulsed dye laser therapy has shown good results in managing superficial vascular malformations, including capillary and glomuvenous malformations [20,21]. Adverse effects are rare and include transient hyperpigmentation, with ulceration and scarring occurring in very few cases. However, laser therapy is ineffective for VMs located deep within tissues, as it would require intraoperative application.

The gold standard for minimally invasive VM treatment is sclerotherapy, which is used to manage symptoms and reduce the volume of extra-articular VMs. It is performed percutaneously or intraoperatively with sclerosing agents such as bleomycin and ethanol [6,15]. Sclerotherapy is a low-risk procedure with a short recovery time, though potential local complications include pain, localized edema, skin necrosis, and ulceration. Misadministration of the sclerosant can lead to systemic complications, including thrombosis, pulmonary embolism, bradycardia, and renal toxicity. Percutaneous sclerotherapy is not recommended for the treatment of IAVMs due to the high risk of cartilage damage from the sclerosing agent. Instead, arthrotomy is the treatment of choice and should be performed as soon as possible after diagnosis [1,6,15]. If left untreated, these malformations typically follow a pattern of remission and relapse, with pain, knee swelling, and eventual joint contracture being the most common symptoms. Arthroscopy is not routinely performed due to its limited field of view and the high risk of intra-articular bleeding. However, some authors suggest that exploratory arthroscopy should be considered in patients with inconclusive MRI findings regarding synovial involvement [2,3]. The use of Mammalian Target of Rapamycin inhibitors (mTOR inhibitors), such as sirolimus, shows promise in treating complex vascular malformations that are not amenable to surgical excision. Orally administered sirolimus has been shown to induce regression of VMs and effectively reduce symptoms in cases where total lesion removal is not feasible [21].

This study has certain limitations. Its retrospective design precluded real-time assessment of patients’ clinical conditions. Additionally, the rarity of IAVM resulted in a small study group, limiting the possibility of conducting robust statistical analyses. Moreover, as a single-center study, our findings and perspective on this pathology may differ from those of other researchers. A multicenter prospective study with well-defined inclusion and diagnostic criteria would provide a more comprehensive understanding of this rare but clinically significant condition, ultimately improving both diagnostic and therapeutic approaches.

## 5. Conclusions

IAVMs are rare causes of knee dysfunction in children and young adults, particularly in cases of unexplained knee pain, swelling, or joint instability. They should be considered in the differential diagnosis of conditions such as hemophilic arthropathy, juvenile idiopathic arthritis, or late sequelae of trauma. Untreated IAVMs can lead to intra-articular bleeding, cartilage degeneration, and knee disability. Early diagnosis using imaging techniques, primarily MRI and ultrasound, is essential for identifying IAVMs and preventing joint degeneration. Timely treatment can help avoid further joint damage and long-term disability.

## Figures and Tables

**Table 1:** Clinical symptoms.

Patient No.	Age	Hemarthrosis	Pain and Swelling	Pain Character	Duration of Pain and Swelling	Aggravating Factors	Deformation of Affected Limb	Contracture of Affected Limb
1	21	−	+	Dull	Chronic	−	+	−
2	10	+	+	Throbbing, stretching	Intermittent	Exertion	+	+
3	20	+	+	Burning	Chronic	−	+	+
4	14	+	+	Bursting, constricting	Chronic	−	+	+
5	8	−	+	Stabbing	Chronic	−	+	−
6	3	+	+	Dull	Intermittent	Exertion	+	+
7	15	−	+	Constricting	Chronic	−	−	−
8	15	−	+	Throbbing	Intermittent	Exertion	+	−
9	22	+	+	Throbbing, squeezing	Intermittent	Exertion	+	+

**Table 2:** Radiological features of VM of the knee on contrast-enhanced MRI.

Patient No.	MRI Sequences Used	Lesion Location	Intra-/Extra-Articular	Extracapsular Structures Involved	Deformation of Affected Limb
1	T1, T2 Fat-Sat, STIR, T2, GRE, T1 Pre-Contrast, T1 Post-Contrast, T1 Post-Contrast Fat-Sat, CE-MRA	Thigh, Hoffa’s fat pad, suprapatellar recess	Both	Subcutaneous tissue, muscles, ligaments	+
2	T1, T2 Fat-Sat, STIR, PD Fat-Sat, GRE, T1 Pre-Contrast, T1 Post-Contrast Fat-Sat, DWI, CE-MRA	Thigh, crus, Hoffa’s fat pad, suprapatellar recess	Both	Subcutaneous tissue, muscles, ligaments	+
3	T1, T2, STIR, T2 Fat-Sat, GRE, T1 Pre-Contrast Fat-Sat, T1 Post-Contrast, MRV, CE-MRA	Gluteal region, thigh, Hoffa’s fat pad, suprapatellar recess	Both	Subcutaneous tissue, muscles, ligaments	+
4	T1, PD, STIR, T2 Fat-Sat, GRE, T1 Pre-Contrast Fat-Sat, T1 Post-Contrast, 3D TOF-MRA, CE-MRA	Thigh, crus, all recesses of knee, Hoffa’s fat pad	Both	Subcutaneous tissue, muscles, ligaments	+
5	T1, T2, STIR, T2 Fat-Sat, GRE, T1 Pre-Contrast, T1 Post-Contrast, DCE-MRI, CE-MRA	Thigh, Hoffa’s fat pad, suprapatellar recess	Both	Subcutaneous tissue, muscles, ligaments	+
6	T1, T2, STIR, T2 Fat-Sat, GRE, T1 Pre-Contrast Fat-Sat, T1 Post-Contrast Fat-Sat, MRV, CE-MRA	Gluteal region, thigh, crus, Hoffa’s fat pad, suprapatellar recess	Both	Subcutaneous tissue, muscles, ligaments	+
7	T1, T2, STIR, T2 Fat-Sat, GRE, T1 Pre-Contrast, T1 Post-Contrast, DTI, CE-MRA	Gluteal region, thigh, crus, foot, Hoffa’s fat pad, suprapatellar recess	Both	Subcutaneous tissue, muscles, ligaments	−
8	T1, T2, STIR, PD Fat-Sat, GRE, T1 Pre-Contrast, T1 Post-Contrast Fat-Sat, DWI, CE-MRA	Crus, Hoffa’s fat pad, suprapatellar recess	Both	Subcutaneous tissue, muscles, ligaments	+
9	T1, T2, STIR, T2 Fat-Sat, GRE, T1 Pre-Contrast Fat-Sat, T1 Post-Contrast Fat-Sat, 3D TOF-MRA, CE-MRA	Hoffa’s fat pad, suprapatellar recess	Intra-articular	−	−

## Data Availability

The original contributions presented in the study are included in the article, further inquiries can be directed to the corresponding author.
